# The Role of Vitamin D in Brain Health: A Mini Literature Review

**DOI:** 10.7759/cureus.2960

**Published:** 2018-07-10

**Authors:** Ibrar Anjum, Syeda S Jaffery, Muniba Fayyaz, Zarak Samoo, Sheraz Anjum

**Affiliations:** 1 Internal Medicine, The University of Texas MD Anderson Cancer Center, Houston, USA; 2 Research, CiBNP, Fairfield, California, USA; 3 Internal Medicine, Fatima Memorial Hospital, Lahore, PAK; 4 Medicine, Ziauddin College of Medicine, Karachi, PAK; 5 Medicine, Sherif Medical City Hospital, Lahore, PAK

**Keywords:** vitamin d deficiency, dementia, depression, diabetes mellitus, autism, schizophrenia

## Abstract

Vitamin D is vital for our body as it regulates calcium homeostasis and maintains bone integrity. In this article, we will discuss how vitamin D aids in the function of neuronal and glial tissue and the many health consequences in a person with vitamin D deficiency. Some of the effects of vitamin D deficiency that will be discussed include the development of dementia caused by the increase of cerebral soluble and insoluble amyloid-β (Aβ) peptides and a decrease of its anti-inflammatory/antioxidant properties, the link to depression by a reduction of the buffering of increased calcium in the brain, and vitamin D deficiency in expecting mothers linking to the development of autism and schizophrenic-like disorders, hypoxic brain injury, and other mental illnesses. Lastly, we will discuss how vitamin D deficiency is linked to the development of diabetes mellitus, its role in neuronal development and a decrease of microglial inflammatory function leading to increased brain infections.

## Introduction and background

Vitamin D a lipid soluble vitamin, also known as sunshine vitamin synthesized in our skin in the presence of sunlight [1]. This vitamin strongly known for its role in calcium and bone metabolism and maintaining bone integrity, has been deduced to have more functions than just that [2]. Scientists with the support of multiple types of research have linked the hormone-like vitamin to some disorders throughout the body such as cardiovascular disease, cancer, stroke, metabolic disorders including diabetes [3]. Cognitive impairment, dementia, psychosis, and autism have been added to the list as well now in the interchange of decreased vitamin D levels [4]. The importance of vitamin D3 in reducing the risk of these diseases continues to rise due to the increasing portion of the population in developed countries having a significant vitamin D deficiency [5]. The older population is at an unusually high risk for vitamin D deficiency due to the decreased cutaneous synthesis, and dietary intake of vitamin D. Vitamin D receptors are widespread in brain tissue, and vitamin D's biologically active form (1,25(OH)(2)D3) has shown neuroprotective effects including the clearance of amyloid plaques, a hallmark of Alzheimer's disease [6]. Recent studies have confirmed an association between cognitive impairment, dementia, and vitamin D deficiency. A growing body of literature also suggests that higher serum 25-hydroxyvitamin D (25(OH)D) concentrations, either in utero or early life, may reduce the risk of autism [7]. Indeed, vitamin D was reported to modulate the biosynthesis of neurotransmitters and neurotrophic factors; moreover, its receptor was found in the central nervous system. Vitamin D deficiency was therefore assessed as a risk factor for the multiple diseases aforementioned [8]. In this review, we will discuss and summarize the current knowledge of vitamin D’s role in improving brain function and the relation of vitamin D deficiency to various central nervous system disorders.

## Review

Vitamin D is an essential micronutrient for bone growth and regulation of calcium homeostasis. It not only plays a vital role in skeletal growth but also has other critical biological actions in neurodevelopment and function. The primary source of vitamin D is sunlight; it is also obtained from a few foods such as oily fish and fortified margarine. Vitamin D carries a cholesterol backbone and has steroid-like effects. It is the fat-soluble hormone that plays an essential role in brain health [9]. Recently vitamin D is increasingly recognized as a necessary neuro-steroid with various actions in the brain [10]. Circulating 25(OH) vitamin D crosses the blood-brain barrier and enters glial cells and neuronal cells to be converted into 1,25(OH) 2 D, which is the active form of vitamin D [11]. In this article, we will discuss the active form of vitamin D, calcitriol which mediates its effects by binding to the vitamin D receptor (VDR), which is principally located in the nucleus of target cells [12]. VDR is a nuclear steroid receptor through which it performs its functions in the brain. It has been found that synthesis and destruction of vitamin D occur in the brain and VDR which is required for vitamin D to show its effect is seen in different regions of the brain.

In the last decades, several studies were conducted which shows the association between vitamin D and brain health and the impact of vitamin D deficiency on the brain. In 2016, Miller BJ et al. conducted a human study which shows that vitamin D increases plasma Aβ in older adults, indicative of decreased brain Aβ [13]. In March 2016, Annweiler C et al. conducted a study which demonstrates the neurosteroid actions of vitamin D in the regulation of calcium homeostasis, β-amyloid deposition, antioxidant and anti-inflammatory properties. It also discusses neuroprotection action of vitamin D against neurodegenerative process associated with Alzheimer’s disease and cognition [14].

A meta-analysis conducted in 2017 shows the hypothesis that 25(OH) D concentrations less than 25 nmol/l increases risk for dementia especially in adults and patients above 65 years of age [15-16]. A study conducted by Durk MR et al. on mice investigates the role of vitamin D3 receptor in reducing cerebral soluble and insoluble amyloid-β (Aβ) peptides. It shows that VDR is a potent therapeutic target in the prevention and treatment of Alzheimer’s disease [17]. Furthermore, a study conducted by Annweiler et al. shows that hypovitaminosis D is commonly seen in adults and 65-year-old patients. They show sign of dementia and cognition impairment [18].

Several studies show the link between vitamin D and depression. A study conducted in 2015 shows the low levels of serotonin in the hippocampus seen in depression [19]. Evidence from animal studies shows the behavioural and anatomical changes in the hippocampus in animals with low vitamin D [20]. Berridge conducted a study in 2017 which have evidenced that depression caused by an imbalance between excitatory and inhibitory pathways in the brain. Hypothesis argues that vitamin D reduces the increase in neuronal levels of calcium (CA +2) that are driving depression. Vitamin D plays a role in maintaining the expression of the CA 2+ pumps and buffers that reduce CA 2+ levels, which may explain how it acts to reduce the onset of depression [21].

In 2014, Gezen-AK et al. conducted a study which shows that vitamin D regulates the release of nerve growth factor (NGF), an essential molecule for the neuronal survival of hippocampal neurons as well as cortical neurons [22]. Kelly L conducted a study which demonstrated that vitamin D insufficiency might relate to higher levels of anxiety and depression, in turn contributing to the elevated risk of psychosis in children with chromosome 22q11.2 deletion syndrome (22q11.2DS). This syndrome is a complex developmental disorder with serious medical, cognitive and emotional symptoms in their lifespan [23].

In early life, vitamin D plays a vital role in neuronal development. Some studies conducted recently shows the effect of vitamin D on early life brain development. In May 2018, a study conducted by Yates et al. shows that deficiency of vitamin D in maternal and offspring shows some disabilities in early life including learning and memory problems and grooming behaviours. There was also some evidence of increased lateral ventricle volume and altered neural expression of genes involved in dopamine and glucocorticoid-related pathways suggesting autism and schizophrenic-like disorders [24]. In March 2018, Freedman et al. performed a systematic review of prenatal nutrients and childhood emotional development and later mental illness. The result shows prenatal nutrients including vitamin A and D are required in pregnancy to decrease the risk for schizophrenia and other mental illness in later life of offspring [25]. Vitamin D plays a vital role in neonatal hypoxic-ischemic brain injury. In February 2018, Stessman et al. conducted a study which demonstrates pregnant mothers are at higher risk for developing vitamin D deficiency. Babies born to vitamin D deficient mothers developed hypoxic-ischemic brain injury [26].

Di Somma et al., in a study, shows optimal levels of vitamin D in the bloodstream are necessary to preserve the neurological development and protect the adult brain [27]. Balanced dietary intake is a well-established lifestyle factor in maintaining cognition during ageing. A recent study shows that vitamin D helps in keeping cognitive function in older adults [28]. An interesting study conducted by Harrison et al. shows the association of vitamin D deficiency and the development of diabetes mellitus through paraventricular hypothalamic nuclei. It shows the positive relationships between them [29]. In November 2017, Kesby JP et al. conducted a study showing the effect of vitamin D on both widespread neurotransmitter changes (glutamine/noradrenaline) and regionally selective neurotransmitter changes (dopamine/serotonin). It concluded that developmental vitamin D deficiency leads to these brain changes [30-31]. Due to its effect on dopamine pathway in the brain, vitamin D can be a useful therapeutic agent used as an intervention therapy to be combined with existing treatments for Parkinson’s disease [32]. Staphylococcal enterotoxin B (SEB) is a superantigen and can initiate inflammation. Microglial cells in brain fight against these types of inflammation. Vitamin D deficiency affects the inflammatory process in the brain causing exposure of the brain to these vulnerable pathogens [33].

## Conclusions

In conclusion, vitamin D is essential to maintain important functions of the body such as calcium homeostasis, maintenance of skeleton integrity, and neurodevelopment. Its deficiency has been linked to many problems such as dementia, depression, diabetes mellitus, autism, and schizophrenia. It is important that this topic is emphasized since correcting the deficiency state can help prevent many negative health consequences.
